# Training clinical professionals to deliver a patient centered intervention in healthcare settings

**DOI:** 10.1186/s12909-024-06151-1

**Published:** 2024-10-13

**Authors:** Monica M. Matthieu, Ciara M. Oliver, Jane Ann McCullough, Mary J. Mallory, Laura D. Taylor, Jennifer A. Koget, Jamie Jensen, David A. Adkins, Robin M. Smith, Kimberly K. Garner

**Affiliations:** 1 U.S. Department of Veterans Affairs Medical Center, Central Arkansas Veterans Healthcare System, HSR & D Center of Innovation, Center for Mental Healthcare & Outcomes Research, 2200 Fort Roots Drive, North Little Rock, AR 72114 USA; 2 Saint Louis University, School of Social Work, 3500 Lindell Blvd, Saint Louis, MO 63103 USA; 3 U.S. Department of Veterans Affairs Medical Center, Central Arkansas Veterans Healthcare System, Geriatric Research, Education and Clinical Center, 2200 Fort Roots Drive, North Little Rock, AR 72114 USA; 4 U.S. Department of Veterans Affairs, National Social Work Program, Patient Care Services, Care Management and Social Work Services, 810 Vermont Avenue, NW Washington DC, 20420 USA; 5 U.S. Department of Veterans Affairs Medical Center, San Francisco Veterans Healthcare System, 4150 Clement Street, San Francisco, CA 94121 USA; 6 College of Medicine, Department of Psychiatry, University of Arkansas for Medical Sciences, 4301 W Markham Street, Little Rock, AR 72205 USA

**Keywords:** Advance Care Planning, Advance directives, Veterans, Group, Veterans Health Administration, Patient-centered care

## Abstract

Within the healthcare settings of the United States Department of Veterans Affairs (VA), one patient-centered intervention, Advance Care Planning via Group Visits (ACP-GV), engages veterans and those they trust in advance care planning (ACP) by facilitating a discussion that encourages participants to plan for future healthcare needs. ACP-GV is a one-hour, single session group intervention facilitated by a trained clinical professional (e.g., physician, nurse, social worker, psychologist, chaplain) and delivered in a healthcare or community-based setting. Using reporting guidelines for group-based and educational interventions, this paper aims to describe the ACP-GV Facilitator Training used to prepare clinical professionals to offer the ACP-GV intervention to participants. We provide health professional students and early career health professionals with an overview of the training and key tips for using group modalities in the clinical setting. Although the training is initially directed towards health professionals who are learning to offer ACP-GV for the first time, our tips for teaching also focus on and extend to facilitating ACP-GV directly with veterans, caregivers, and those they trust. The ACP-GV Facilitator Training is sequential in that it expects clinicians to first learn the required educational content and how to plan a group, then it engages clinicians in practicing group facilitation skills. At the conclusion of the training, clinicians are then instructed to use the training materials to transfer the information and skills they learned about ACP-GV to patients they encounter in their respective work settings. The culmination of the ACP-GV Facilitator Training is, therefore, when the clinician is able to facilitate their own group, guide discussions and activities, actively use training materials, and encourage veterans and those they trust to participate in a discussion regarding ACP in a group setting. Finally, we share key resources for publicly available and accessible online trainings to promote spread outside of VA. ACP-GV’s Facilitator Training can assist healthcare professionals in implementing ACP-GV in a variety of care settings.

## Introduction

Advance care planning (ACP), mandated by the Patient Self Determination Act [1] and implemented in healthcare settings within the United States Department of Veterans Affairs (VA) [2], is a discussion based on a veteran’s values and preferences concerning their future care. ACP engagement increases the likelihood that an individual’s healthcare wishes will be followed [3]. It is associated with less in-hospital death, a greater likelihood of hospice use, and can reduce caregiver stress, depression, and anxiety [3, 4]. An Advance Directive (AD) form is an optional, albeit important, part of ACP that documents care preferences for an individual’s friends, family, and care team to follow if they are unable to direct their own care.

Advance Care Planning via Group Visits (ACP-GV) is an innovative approach to ACP using a group intervention to discuss care preferences and options amongst group participants. In this paper, we aim to describe an educational training developed by the National ACP-GV Program for dissemination within VA healthcare facilities and beyond. We outline the description using a standardized methodology checklist designed to improve reporting of group-based behavior-change interventions [5] and the Guideline for Reporting Evidence-based practice Educational interventions and Teaching (GREET) 2015 checklist [6]. First, we provide health professional students and early career health professionals with an overview of the ACP-GV Facilitator Training, including the historical and theoretical background, then we describe the materials and educational content necessary to deliver a single session ACP-GV group. Within the ACP-GV session, key concepts that are integral to delivering the model with fidelity are outlined, then key terms are defined. Finally, we end with additional tips for training prospective group facilitators who intend to offer ACP-GV broadly in their respective VA and/or non-VA healthcare settings.

## What is ACP-GV?

### Intervention overview

ACP-GV is a patient-centered, group-based, educational intervention that engages patients and those they trust in a 60-minute discussion about ACP. The intervention elicits personal experience(s) and encourages participants to identify a ‘next step’ in planning for future care needs. The objective of ACP-GV is to increase each participant’s motivation to engage in ACP. Group size may vary, and groups can be facilitated virtually or in-person by an ACP-GV trained clinician (e.g., physician, nurse, social worker, psychologist, chaplain). Small groups of two or three individuals are common and it is recommended that groups do not exceed ten participants. In VA healthcare settings, ACP-GV is offered to any healthcare beneficiary receiving VA care in inpatient, outpatient, residential, or community-based care setting, regardless of their health status, that is participating in the National ACP-GV Program. To date, the National ACP-GV Program has been implemented in 75 of 152 VA facilities across the nation [7].

### Intervention source and development methods

In 2013, Dr. Kimberly Garner and VA colleagues sought to deliver ACP educational content to veterans in a group setting. The resulting program, ACP-GV, has garnered acclaim within VA as an Office of Rural Health (ORH) Promising Practice, a Gold Status Practice by the VA Diffusion of Excellence Initiative, and became an ORH-funded Enterprise-Wide Initiative in July 2019. This initiative funded a national ACP-GV team to increase access and engagement in ACP by veterans living in rural areas who typically receive their primary care in a VA community-based outpatient clinic setting. This rural focus, coupled with the diversity, equity, and inclusion initiative for VA facilities in urban areas, spawned a National ACP-GV Program that developed a publicly accessible ACP-GV Facilitator Training. This funding allowed the ACP-GV Facilitator Training to be offered nationally for instructing interested clinicians, health professional students, and early career health professionals on how to facilitate and lead an ACP-GV group.

### Theoretical and evidence-based concepts

To ensure consistency of ACP-GV facilitation across the nation, expert VA healthcare staff (including a Geriatrician, Social Workers, and a nurse) developed the standardized training. Their goal was to teach other VA health professional students, early career professionals, and existing clinical staff not only how to facilitate ACP using group modalities, but also how to integrate ACP-GV as a patient-centered intervention into the culture of the healthcare system. These experts, along with a curriculum specialist, developed and refined the ACP-GV Facilitator Training using the Successive Approximations Model of Instructional Design [8]. This model is an iterative approach to e-learning that ensures the content is suitable for adult learners and relevant to veterans in various care settings [9].

#### Change mechanisms and theories of change

Interspersed throughout the ACP-GV Facilitator Training, facilitators are taught to intentionally utilize Motivational Interviewing (MI) principles, skills, and techniques during the group [10]. The application of MI is to increase the participant’s own motivation for change and empower them to take charge of their health and well-being by engaging in ACP discussions and actions [11]. Facilitators are also taught the synchronicity of MI with the Transtheoretical Model of change, most commonly referred to as the stages of change model [12], and learn to recognize that various levels of readiness to change exist in each individual. In this model, decision-making regarding a health behavior change follows a progression. It begins with precontemplation, the first stage in which an individual is not considering change, then to contemplation, the second stage in which the individual may be seriously considering a change. Next, it moves to preparation, the third stage in which planning and commitment to change takes place and then to action, the fourth stage in which the individual is taking specific behavioral steps to change. Finally, the individual arrives at the last stage, called maintenance, in which they are working to sustain the changes they have made [12]. Should maintenance fail, often referred to as ‘relapse’ within substance use treatment, the individual seeking change cycles back to previous stages.

## Intervention content

### Activities during the session

ACP-GV facilitators learn to lead two activities during the session: (1) a large group discussion and (2) a written activity. For the large group discussion, the facilitator encourages participants to engage in open discussion by sharing personal experience(s). The written activity includes filling out a document (see below) at the start and end of the group. However, participants may choose to just listen during the discussion and not complete the document.

### Materials

**Fig. 1:**
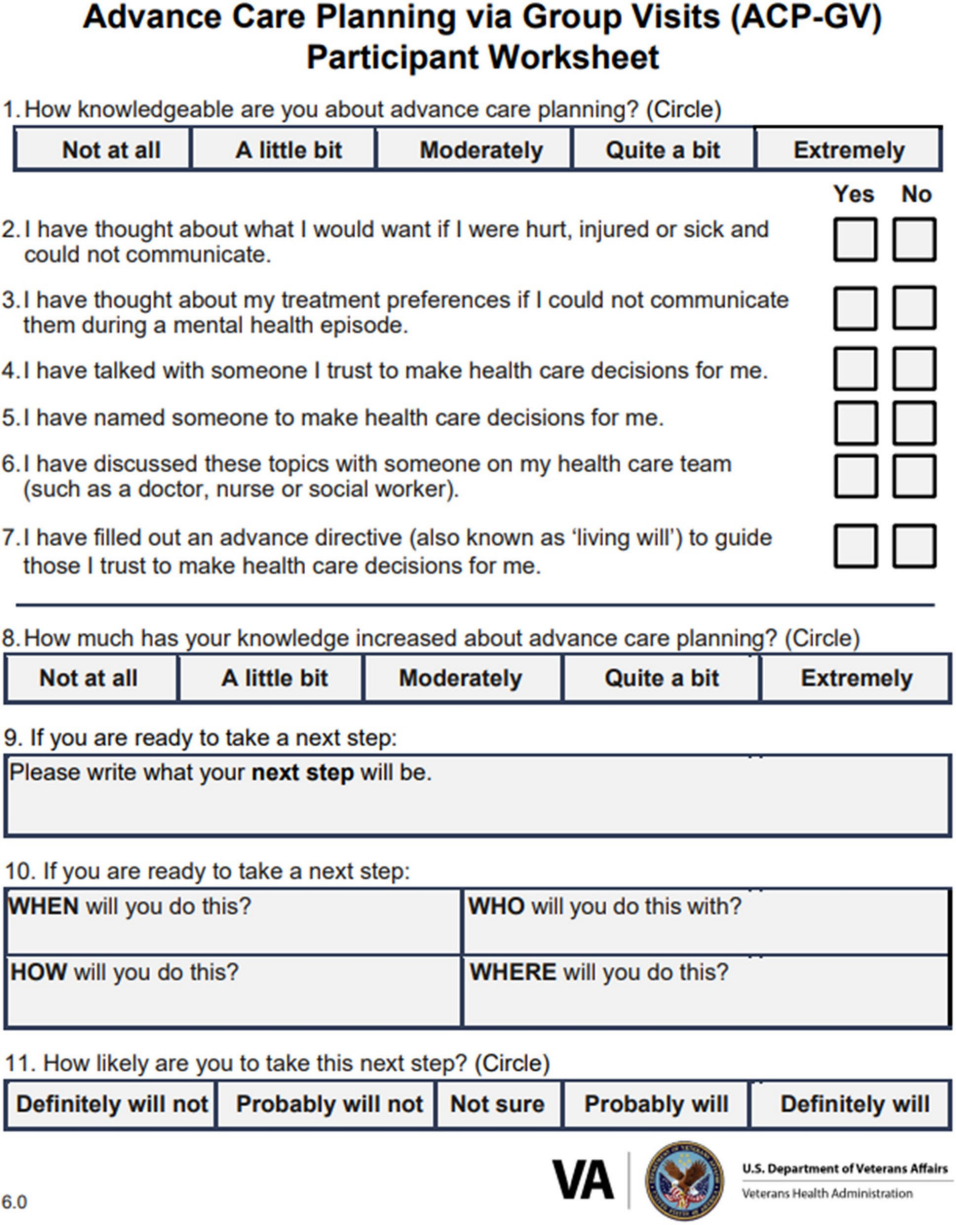
Advance care planning via group visits worksheet

Facilitators use two different required documents as tools during the group. First, the ACP-GV Worksheet (See Fig. 1 below) is used to elicit participant responses to questions regarding their prior knowledge and experience with ACP and then, at the end of group, queries participants about their knowledge gain upon completion. The worksheet is available in large print and screen-reader compatible format. Secondly, a copy of the official document used to execute an AD in the VA healthcare system are provided to veteran participants [13]. Lastly, although optional, it is recommended to provide non-veteran participants with a paper or electronic resource for the format and requirements for each state’s AD for their personal or family ACP use and to encourage documentation of their preferences in an AD.

### Structure of group/session content

**Fig. 2:**
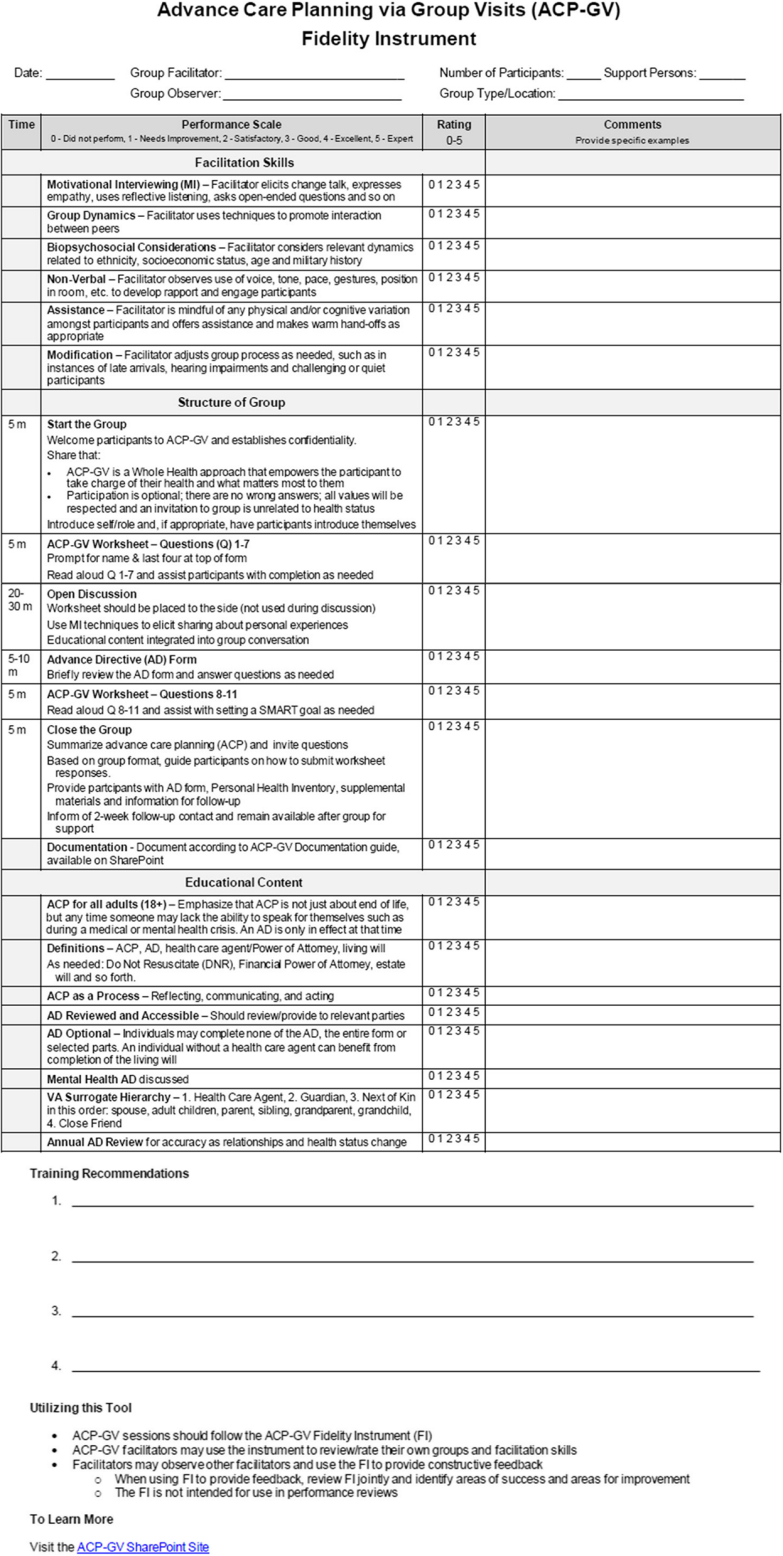
Advance care planning via group visits fidelity instrument

The facilitator begins an ACP-GV session with a welcome and brief introduction of group participants, to include veterans, caregivers, and those they trust, ensuring participants respect limits of confidentiality and shared perspectives, and to confirm participants understand participation is optional. Next, the facilitator introduces the ACP-GV Worksheet (See Fig. 1) to participants with instructions to complete questions 1–7 before placing their worksheet to the side. Then, the facilitator guides the group in an open discussion about ACP key concepts and, in the process, defines some key terms and explicates their usage. Once this educational content is complete, the facilitator prompts the group to review the second half of the ACP-GV Participant Worksheet, questions 8–11. Then, the facilitator leads the group through an activity to create a SMART goal (a mnemonic acronym for specific, measurable, achievable, realistic, and timely) [14] which is referred to as a ‘next step’ on the worksheet. Lastly, the facilitator closes the group with a summary of topics discussed, allows time for questions, provides additional forms and resources for participants to take with them, and informs them to expect to be contacted approximately two weeks after group to follow-up. The core components (e.g., facilitation skills, group structure, and educational content) for the ACP-GV model is outlined in Fig. 2 (see below) in the ACP-GV Fidelity Instrument completed by facilitators at the end of group.

### Key concepts

Facilitators introduce a variety of key concepts that are used to deliver specific educational content that is intentionally aligned with the stages of behavior change [12]. The key concepts are process based topics that include reflecting on life experience, learning ACP terms, reflecting on personal values, qualities of a decision-maker, and lastly, documenting preferences. Facilitators weave the key concepts into the educational content that must be covered within the structure of the group discussion (See Fig. 2).

#### Reflecting on life experience

The facilitator introduces the first key concept, reflecting on life experience, which allows participants to speak openly about their personal and familial experiences with ACP, increasing understanding by group participants, perception of relevance to the topic of ACP, and feelings of self-efficacy as a participant in the group. The group conversation also helps participants connect emotionally to the topic of ACP, and during this time, the facilitator educates participants that ACP is a renewable, accessible process for adults 18 and older, and not only performed at end of life. Next, facilitators emphasize that ADs are only used when patients are unable to direct their care. This means that the ADs only go into effect when the patient is incapacitated (e.g., medically induced coma due to accident). When cognitive capacity is regained, the patient resumes decision-making. Furthermore, ADs can also be used as a planning document for future care in the case of an upcoming procedure, surgery, or just during a routine AD review.

#### ACP terms

Next, the facilitator teaches participants commonly used VA key terms and definitions for ACP [2]. The three most important terms include AD, Healthcare Power of Attorney, and Living Will. An AD is a legal document that contains both the Healthcare Power of Attorney section and the Living Will section. The Healthcare Power of Attorney (also referred to as “healthcare agent”) is an individual that the participant trusts to represent them if they become incapacitated, and the Living Will allows them to provide specific guidance about circumstances in which they may or may not want life-sustaining treatments provided. As they become more educated on ACP, facilitators can also discuss other terms, such as a do not resuscitate orders, Financial Power of Attorney, and estate wills. However, many health professional students, early career health professionals, and experienced clinicians may need additional time and training on these terms. It is recommended that all clinical staff planning to conduct ACP-GV review organizational policies and ethical guidelines for their respective healthcare system. A VA healthcare organizational policy with relevant key terms and definitions are provided at https://www.ethics.va.gov/docs/policy/ACP_Policy.pdf .

#### Reflecting on values

The facilitator then encourage participants to reflect on their values and guides the group in conversation through a process of reflecting, communicating, and documenting what their care team and those they trust need to know if they become incapacitated. This reflection, focusing on values, deepens the conversation around those core ideals that guide their life, decisions, and perspectives. From this discussion, the facilitator encourages participants to consider how to translate these values to action. Identifying clear preferences can then evolve into specific steps to be taken if the veteran becomes incapacitated or unable to communicate. The AD, which is optional, can include any information they feel is pertinent to the care they may receive, the handling of their belongings, communicating with family, or even the care of beloved pets. A separate AD that focuses on mental health is also offered. For individuals without a healthcare agent, providing clear instructions for the use of a Living Will is offered as an option. These action steps and options provide the valuable basis for communicating preferences and direction for loved ones to step in to assist in the veteran’s care plans when, and if, necessary.

#### Qualities of a decision-maker

Next, the facilitator discusses qualities of a decision-maker to open the conversation about the VA surrogate hierarchy. Some of the essential qualities include someone who is emotionally grounded, comfortable in a healthcare setting, and familiar with and able to honor the participant’s values. Following this identification of an individual with these qualities, the facilitator encourages participants to have an ACP discussion with their healthcare agent, share their treatment preferences, and confirm they are comfortable serving in this role. Finally, facilitators urge participants to have additional ACP conversations to ensure that relevant trusted others are aware of the healthcare agent’s role, especially if the agent is not a family member.

After the initial process of eliciting qualities, the next educational content woven into the discussion is education on the surrogate hierarchy order. This hierarchy is an important aspect of the training in VA healthcare settings, as it will be used to guide VA as an organization if the veteran does not have a Healthcare Power of Attorney appointed. This guidance allows the VA healthcare team to determine who would be the decision-maker for the veteran in the event the veteran is incapacitated. The facilitator informs the group the order is, (1) Spouse, (2) Adult children, (3) Parents, (4) Siblings, (5) Grandparents, (6) Grandchildren, and (7) Close friend. This hierarchy is especially important for individuals who are not legally divorced but separated to note that spouses would be asked by VA to be the veteran’s decision maker. Other case examples are for unmarried individuals without children or siblings, the decision maker may be a close friend, as parents or grandparents may be deceased. From this example, it is easy to see how the lack of a healthcare agent can impede the clinical team in identifying suitable representatives to coordinate care for the patient.

#### Documenting preferences

The last key concept is documenting preferences. At this point, the facilitator directs the participants to paper or electronic versions of the VA AD. All other participants are directed to the AD requirements governed by their respective state law. The facilitator reviews the VA AD form section by section, reading aloud, discussing critical areas not to be overlooked, and provides copies for group participants. Some particular areas to highlight include the veteran’s ability to complete the Durable Power of Attorney for Health Care, to include identifying a primary and an alternate Health Care Agent. The participants are shown how to fill out this section of the form before moving onto the Living Will section. The Living Will section of the AD presents the veteran with different healthcare scenarios to review and allows them to choose their treatment preferences for each scenario, to include life-sustaining treatments. Then, participants review the Mental Health Preferences section of the AD. This section is especially important for veterans living with a serious mental illness and can allow them to describe their preferences for mental health treatments. The Additional Preferences section is the last section reviewed, and participants are encouraged to use this section for additional and miscellaneous items, like preferred medications, care of their pets, or anything relevant to their care if they become incapacitated. Finally, the review of ADs is noted, with recommendations to update ADs annually or when key life events occur related to marriage, divorce, and/or deaths in the family.

## Intervention design

### Sequencing of sessions

ACP-GV is a one-session group following the same content, structure, and model for each offering facilitated by a clinician. However, while not a formal part of the group, the intervention also recommends facilitators follow-up via telephone, virtual, or in person approximately two weeks after group participation to answer any remaining questions and to encourage taking the next step that each participant identified for themselves as part of the ACP-GV Participant Worksheet in group.

### General setting

As previously noted, ACP-GV is delivered in VA healthcare and/or community-based settings. Within ACP-GV Facilitator Training, clinicians are taught a few considerations about the types of groups, venue characteristics, and scheduling that are worth noting. In healthcare settings, there may be two different types of groups. The first type of group is a ‘stand-alone’ group, created and coordinated by ACP-GV staff for the sole purpose of providing ACP discussions in a group setting. In the second type of group, ACP-GV is offered as one installment within the established structure of an unrelated group series, such as groups in mental health, whole health (e.g., wellness, health promotion, and disease prevention), substance use recovery, or other multi-session group formats. Since the ACP-GV intervention is portable, it has been successfully integrated into already established groups such as these. While both group types have merit, time and resources (such as a meeting room) are needed for planning stand-alone ACP-GV groups. It is important facilitators building relationships with leaders of ongoing groups that are delivered in outpatient, inpatient, and residential care settings to ensure opportunities are available to offer ACP-GV in these settings.

### Venue characteristics

When planning in-person groups, it is best for group facilitators to secure adequately private and quite conference rooms with a large conference table and chairs. This type of venue and furniture set up allows for open, approachable discussion among participants and a hard writing space for review and completion of group materials. Facilitators have also held groups in community-based settings such as places of worship, public libraries, and other non-healthcare venues that have space and availability. For virtual groups, VA’s secure telehealth platform, VA Video Connect, is used which connects the group facilitator via their computer and internet connection to participants in varied private, quiet locations, such as their home or vehicle. For participation in VA Video Connect, participants may use a capable smart phone, a home computer, or tablet. Since participants in a virtual group are participating from multiple different physical locations, securing a venue is not necessary.

### Schedule

#### Length of group session

Facilitators scheduled 60 min for an ACP-GV session. While this length of time includes open discussion and addressing participant questions, facilitators may want to allot extra time in their schedules after group concludes to address any referral needs or coordinate follow-up requests.

#### Frequency of group sessions

The frequency of ACP-GV offerings and varies from setting to setting, based on demand, referrals, inpatient or residential census, and availability of time, technology, space, and trained facilitators. The unique needs of the participants and the setting (e.g., clinic, patient, and staffing workflow) should be considered when determining group frequency (e.g., weekly, biweekly, monthly), modality (e.g., in person, virtual) and format (e.g., open or closed group).

#### Duration of group sessions

ACP-GV is meant to be attended once; however, veterans and those they trust are welcome to attend more than once to feel comfortable with their care choices. Some reasons veterans may attend multiple sessions include to ask additional questions after completing initial ACP steps, returning with a support person or potential healthcare agent, a life transition or change in health status has prompted a need for more ACP education, or to continue attending ACP-GV to offer a perspective they want to share in support of their veteran peers in the group [15].

## Participants

### Group composition

In VA healthcare settings, veterans and those they trust are invited to participate in ACP-GV. The veteran participants are typically enrolled beneficiaries of the VA healthcare system, while others are guests. However, efforts to provide ACP-GV to both veteran and non-veteran participants in other healthcare and community-based settings offer the opportunity to gather veterans with a broader constellation of potential participants. In summary, the group is inclusive of adults 18 and older, regardless of health status or other statuses.

### Methods for group allocation and incentives

Participants are not allocated to groups and can choose in person or virtual modalities, as available, in a setting that offers ACP-GV. There are no incentives or reimbursements provided to participants for attending ACP-GV, as it is offered as part of routine clinical care within VA.

## Facilitators

### Number of facilitators

At least one trained facilitator is required to deliver the ACP-GV session to participants. Co-facilitators are optional. In VA, each healthcare facility is encouraged to have an ACP-GV team consisting of multiple group facilitators so ACP-GV can be offered across different units (e.g., inpatient and outpatient), departments (e.g., surgery, behavioral health, primary care, specialty care and residential care programs), and clinics (e.g., community-based Vet Centers) that comprise the full complement of veterans’ healthcare services offered in the local area.

### Continuity of facilitators’ group assignment

Since ACP-GV is a single session, after training in the model, the ACP-GV group facilitator may offer as many groups as they would like and use a combination of different group configurations. For example, one facilitator’s ACP-GV schedule may include: [1] an open “drop in” group set for a standing time and location weekly [2], a group that includes participants who are scheduled in advance to coordinate with other appointments bringing them onsite to the facility, and/or [3] a group that is offered in conjunction with an unrelated but established and ongoing group elsewhere in the medical center (e.g., Whole Health or recovery groups). The continuity of their assignment to ACP-GV is dependent on their role, their schedule, and the desired frequency of offering the group. If there is a team-based approach to offering groups in their setting of care within the healthcare facility or across a healthcare system, that would allow for additional options for scheduling and engaging a cadre of trained ACP-GV facilitators.

### Facilitators’ professional background

ACP-GV groups are a patient-centered intervention delivered by health professional students, early career health professionals, and/or existing healthcare providers trained in clinical patient interactions. Accordingly, ACP-GV requires clinical staff to facilitate the group. The determination of which types of employees are certified or licensed as healthcare practitioners or clinical staff is aligned to VA’s Human Resources categorization of the professions. ACP-GV is suitable for use with trainees and, as a teaching hospital, VA healthcare policy notes that all trainees from associated health professions must be appropriately supervised [16].

### Facilitators’ personal characteristics

Specific personal characteristics required include being an adult 18 years and older with the appropriate education level commensurate with the health professional degree, certification, or license that they hold to practice as a clinician in VA healthcare settings.

### Facilitators’ training in intervention delivery

Group facilitators must attend a three-hour training on how to deliver ACP-GV. The training is offered to VA healthcare staff as a live, instructor-led online course and as an asynchronous, self-paced online course. The ACP-GV Facilitator Training [15] is comprised of three modules. Module 1 consists of a video where actors role play participating in ACP-GV, Module 2 provides an orientation to the ACP-GV model and outcomes, and Module 3 provides education on the application of MI principles and techniques in group settings. The online asynchronous training is also available for non-VA clinicians from a free, publicly available national learning network called TRAIN [15].

The ACP-GV Facilitator Training is sequential in that it asks clinician to first learn how to plan a group, the required educational content, and then it engages them in practicing group facilitation skills. At the conclusion of the training, clinicians are then instructed to use the training materials to transfer the information and skills they learned about ACP-GV and use it in real-life, on the job with patients. The culmination of the training is, therefore, when the clinician is able to facilitate their own group, guide discussions and activities, actively use training materials, and encourage veterans and those they trust to participate in a discussion regarding ACP in a group setting.

### Facilitators’ materials

Materials provided to facilitators during ACP-GV Facilitator Training include the ACP-GV Fidelity Instrument, the ACP-GV Participant Worksheet, and VA’s Advance Directive. Educational materials are available on a VA internal website [17].

### Intended facilitation style

As noted previously, the group facilitation style is open, approachable, discussion-oriented, and patient-centered. In addition to this style, a number of facilitation skills are noted in the Fidelity Instrument (See Fig. 2). Throughout the discussion of the methods of ACP-GV, we have provided details as to the development of ACP-GV utilizing the theoretical model of the stages of change and the spirit of MI. Given this foundation, the facilitators utilize a variety of MI strategies to engage participants in a group discussion and provide education about ACP, which is discussed in detail elsewhere [11]. Additionally, ACP-GV facilitators are reminded to pay attention to group dynamics, consider the impact of biopsychosocial factors, and their own expression of nonverbal behaviors. Finally, facilitators remain flexible to ensure the group is accessible and accommodating for all participants.

### Limitations, accommodations, modifications, and adaptations

Veterans have various strengths and abilities, and ACP-GV is suitable for most veterans. However, individuals with hearing, visual, or physical limitations may require accommodations, adaptive equipment, or other assistance to fully participate in the group setting. The group worksheet is available in large print and screen-reader compatible format. The group model should not be modified and veterans not recommended for ACP-GV include those similarly not recommended for group psychotherapy, to include individuals in crisis states related to psychosis, substance use, bipolar disorder, and suicidal ideation [18]. ACP can be delivered to these veterans individually.

## Documentation after ACP-GV

### Recording group participation

The National ACP-GV Program has standardized documentation practices for ACP-GV and provides facilitators with clear, step-by-step instructions to ensure consistency within the electronic health record. The national documentation guidance includes the following required components. First, a clinical reminder dialogue, launched in FY 2018, serves as a national note template and was created to standardize the clinical content for each ACP-GV participant’s progress note. Each note must use the ACP-GV clinical reminder dialogue. Secondly, clinicians must document ACP-GV notes using the “Advance Directive Discussion” note title in the Computerized Patient Record System. Thirdly, the National ACP-GV Program requires that all adopting VA facilities build necessary infrastructure by completing a clinic set-up process [19] that established a clinic (or ‘visit location’) specifically for ACP-GV. National guidance outlines how to work with the local informatics staff to build a clinic and add necessary identification codes to flag the clinic so that workload can be identified as ACP-GV and tracked across the enterprise. Lastly, clinical staff are encouraged to develop referral and recruitment processes with various programs, clinics, and/or departments that are consistent with the culture of the facility to educate others on the availability of group.

### Methods for checking fidelity of delivery

The ACP-GV Fidelity Instrument is used in training for health professional students, early career health professionals and/or existing healthcare providers who are new ACP-GV facilitators to educate them on the group structure and content and to help ensure clinician adherence to the model by rating their own group facilitation skills. Additionally, during training, the instrument is used as a checklist during an observation of other facilitators conducting ACP-GV to indicate content covered and to offer feedback on ACP-GV delivery.

### Final thought tips for teaching ACP-GV

The ACP-GV Facilitator Training includes facilitated dialogue between participants about all aspects of ACP, educational components, key concepts, goal setting, and even includes a supportive follow-up contact for all participants. Throughout the group, facilitators help participants reveal what matters most to them [20] and teach communication strategies to translate values and preferences into conversations with those they trust and their healthcare team.

## Conclusion

Proactively preparing for future health care allows veterans of all ages and health statuses to ensure their care aligns with their values, even when they cannot speak for themselves. ACP-GV is an innovative approach that encourages and equips veterans to identify what matters most and to have meaningful conversations with loved ones. This enables loved ones and healthcare teams to honor their healthcare wishes at a future time when the person is unable to communicate and, arguably, at their most vulnerable. Health professional students and early career health professionals can champion the spread of ACP-GV beyond VA and veterans so that it can reach a broader population of individuals seeking care in a variety of healthcare and community-based settings.

## Data Availability

No datasets were generated or analysed during the current study.

## Abbreviations

ACP: Advance Care Planning
ACP-GV: Advance Care Planning via Group Visits
AD: Advance Directive
FY: Fiscal year
GREET: Guideline for Reporting Evidence-based practice Educational interventions and Teaching
MI: Motivational Interviewing
ORH: Office of Rural Health
SMART: Specific, Measurable, Achievable, Realistic, and Timely
VA: U.S. Department of Veterans Affairs

## References

[1] Levin RepSM. Patient Self Determination Act. H.R. 5067–101st Congress Nov 5. 1990. https://www.congress.gov/bill/101st-congress/house-bill/5067#:~:text=Patient%20Self%20Determination%20Act%20of%201990%20%2D%20Amends%20titles%20XVIII%20(Medicare,State%20law%20to%20make%20decisions.

[2] U.S. Department of Veterans Affairs. Advance care planning and management of advance directives. 2023 [cited 2021 Aug 12]. VA National Center for Ethics in Health Care. https://www.ethics.va.gov/docs/policy/ACP_Policy.pdf.

[3] Bond WF, Kim M, Franciskovich CM, Weinberg JE, Svendsen JD, Fehr LS, et al. Advance Care Planning in an Accountable Care Organization is Associated with increased Advanced Directive documentation and decreased costs. J Palliat Med. 2018;21(4):489–502. 29206564 10.1089/jpm.2017.0566 PMC5867515

[4] Bischoff KE, Sudore R, Miao Y, Boscardin WJ, Smith AK. Advance Care Planning and the quality of end-of-Life Care in older adults. J Am Geriatr Soc. 2013;61(2):209–14. 23350921 10.1111/jgs.12105 PMC3760679

[5] Borek AJ, Abraham C, Smith JR, Greaves CJ, Tarrant M. A checklist to improve reporting of group-based behaviour-change interventions. BMC Public Health. 2015;15(1):963. 26403082 10.1186/s12889-015-2300-6 PMC4583168

[6] Phillips AC, Lewis LK, McEvoy MP, Galipeau J, Glasziou P, Moher D, et al. Development and validation of the guideline for reporting evidence-based practice educational interventions and teaching (GREET). BMC Med Educ. 2016;16(1):237. 27599967 10.1186/s12909-016-0759-1 PMC5011880

[7] Matthieu MM, QUERI – Quality Enhancement Research Initiative. 2021 [cited 2022 Dec 20]. Evaluation of the National Implementation of the VA Diffusion of Excellence Initiative (DEI) on Advance Care Planning (ACP) via Group Visits (ACP-GV). https://www.queri.research.va.gov/centers/AdvanceCare.pdf.

[8] Allen MW, Sites RH. American Society for Training and Development. Leaving ADDIE for SAM: an agile model for developing the best learning experiences. Alexandria, Virginie: American Society for Training & Development; 2012.

[9] Allen Interactions Inc. SAM (The Successive Approximations Model) for eLearning Development. 2024 [cited 2024 Sep 24]. https://www.alleninteractions.com/services/custom-learning/sam/elearning-development.

[10] Rollnick S, Miller WR. What is motivational interviewing? Behav Cogn Psychother. 1995;23(4):325–34. 10.1017/S1352465809005128 19364414

[11] Matthieu MM, Oliver CM, Hernandez GI, McCullough JA, Adkins DA, Mallory MJ, et al. Application of motivational interviewing to group: teaching advance care planning via group visits for clinical professionals. Patient Educ Couns. 2024;120:108116. 38150951 10.1016/j.pec.2023.108116

[12] Prochaska JO, Velicer WF. The Transtheoretical Model of Health Behavior Change. Am J Health Promot. 1997;12(1):38–48. 10170434 10.4278/0890-1171-12.1.38

[13] U.S. Department of Veterans Affairs. VA advance directive durable power of attorney for health care and living will. 2021. https://www.va.gov/vaforms/medical/pdf/VA_Form_10-0137_FILL.pdf.

[14] Doran GT. There’s a S.M.A.R.T way to write management’s goals and objectives. In: American Management Association [Internet]. AMA Forum. 1981 [cited 2023 Jan 25]. 35–6. (Management Review; vol. 70). https://community.mis.temple.edu/mis0855002fall2015/files/2015/10/S.M.A.R.T-Way-Management-Review.pdf.

[15] U.S. Department of Veterans Affairs. VHA TRAIN. 2022 [cited 2022 Jun 1]. ACP-GV Facilitator Training - Complete Series (Enduring). https://www.train.org/vha/course/1102553/.

[16] U.S. Department of Veterans Affairs. Department of Veterans Affairs. 2015. Supervision of associated health trainees. https://www.va.gov/OPTOMETRY/docs/VHA_Handbook_1400-04_Supervision_of_Associated_Health_Trainees_03-19-2015.pdf.

[17] Veterans Health Administration. National Advance Care Planning via Group Visits (ACP-GV) Program. 2022 [cited 2022 Jun 10]. VA SharePoint. https://dvagov.sharepoint.com/sites/VHAadvance-care-planning-via-group-appointments.

[18] Ronconi JM, Shiner B, Watts BV. Inclusion and exclusion criteria in Randomized controlled trials of psychotherapy for PTSD. J Psychiatr Pract. 2014;20(1):25–37. 24419308 10.1097/01.pra.0000442936.23457.5b

[19] National ACP-GV, Program, Advance Care Planning via Group Visits (ACP-GV). Clinic Set-Up & Documentation Protocol [Internet]. Department of Veterans Affairs; 2022 [cited 2022 Jun 10]. https://dvagov.sharepoint.com/:w:/r/sites/vhaadvance-care-planning-via-group-appointments/_layouts/15/Doc.aspx?sourcedoc=%7BFA633869-48EC-4E35-93CA-868225CEE776%7D&file=Clinic%20Set-up%20%26%20Documentation%20Protocol%204-2022.docx&action=default&mobileredirect=true.

[20] Matthieu MM, Church KA, Taylor LD, Oliver CM, McCullough JA, Adkins DA et al. Integrating the Age-Friendly Health Systems movement in Veterans Health Administration: National Advance Care Planning via Group visits and the 4Ms Framework. Health Soc Work. 2023;hlad022:1–4. 10.1093/hsw/hlad022 37608558

